# Rat Macrophage C-Type Lectin Is an Activating Receptor Expressed by Phagocytic Cells

**DOI:** 10.1371/journal.pone.0057406

**Published:** 2013-02-28

**Authors:** Ana Lobato-Pascual, Per Christian Saether, Maria K. Dahle, Peter Gaustad, Erik Dissen, Sigbjørn Fossum, Michael R. Daws

**Affiliations:** 1 Department of Anatomy, Institute of Basic Medical Sciences, University of Oslo, Oslo, Norway; 2 Section of Immunology, Norwegian Veterinary Institute, Oslo, Norway; 3 Institute of Clinical Medicine, University of Oslo, Oslo, Norway; 4 Department of Microbiology, Oslo University Hospital, Oslo, Norway; Institute of Microbial Technology, India

## Abstract

Macrophage C-type lectin (MCL) is a membrane surface receptor encoded by the Antigen Presenting Lectin-like gene Complex (APLEC). We generated a mouse monoclonal antibody for the study of this receptor in the rat. We demonstrate that rat MCL is expressed on blood monocytes and neutrophils, as well as on several tissue macrophage populations, including alveolar and peritoneal cavity macrophages. We also demonstrate MCL expression on a subset of resident spleen macrophages. Immunohistochemistry analysis of the spleen showed staining specifically in the marginal zone and red pulp. Exposure to pro-inflammatory mediators or to yeast cell wall extract (zymosan) increased surface MCL expression on peritoneal macrophages. We characterized a rat myeloid cell line, RMW, which expresses high levels of MCL. We found that MCL co-immunoprecipitated with the activating adaptor protein FcεRIγ in these cells. Moreover, beads coated with anti-MCL antibody increased phagocytosis in the RMW cells. Together, these observations indicate that rat MCL is a receptor that activates phagocytosis in myeloid cells under inflammatory conditions.

## Introduction

The gene complex APLEC (Antigen Presenting LEctin-like Complex) was first described by Flornes et al. as a gene cluster located on rat chromosome 4, mouse chromosome 6 and human 12p13 [1]. The complex consists of seven related C-type lectin receptor genes, namely, Dendritic Cell Activating Receptor (DCAR), Dendritic Cell Inhibitory Receptor 1, 2, 3 and −4 (DCIR), Macrophage C-type lectin (MCL), and Macrophage inducible C-type lectin (Mincle). An eighth gene, Dectin-2, is present as a pseudogene in the rat strains examined thus far.

MCL is a type II transmembrane protein with a single extracellular C-terminal C-type lectin-like domain. This domain contains an evolutionarily conserved folded domain, and a carbohydrate recognition domain containing the Ca^2+^ binding sites that give name to this family of proteins [2]. Its presence suggests a possible carbohydrate binding function, although such receptors are also known to recognize protein ligands. Two of the APLEC receptors; Dectin-2 (human) and Mincle (mouse), have been shown to recognize carbohydrate moieties from fungi, yeast, platyhelminthes, house dust mites and bacteria [3]–[8]. C-type lectins are functionally diverse. Their presence on the surface of immune cells and their potential for recognizing polysaccharide structures suggests a central role as pattern-recognition receptors in the innate immune system.

Despite the growing amount of data describing expression and function of the APLEC receptors, very little has been reported about MCL in general, and the rat MCL in particular. The receptor was originally cloned and described in mouse studies as a C-type lectin with macrophage-restricted expression [9], [10], and later in human studies as a macrophage surface receptor that elicits endocytosis when cross-linked on transfected 293T cells [11]. MCL mRNA transcript levels were detected in the bone marrow, peripheral blood lymphocytes, resident peritoneal macrophages, and at a lower level in the spleen and lung. Our group’s earlier work on the APLEC receptors detected expression of MCL transcripts in macrophages, neutrophils, B cells, dendritic cells, and traces in CD4**^+^** T cells. Studies of the human MCL have been hampered by the fact that it does not express readily on the surface of transfected cells, but it is retained intracellularly, suggesting that additional partner molecules are required for assembly of a functional MCL receptor complex. However, recent work using chimeric receptors has demonstrated that MCL is capable of inducing phagocytosis, cytokine production and oxidative burst, suggesting an activating role for this protein [12]. The data we present here agree with the findings of Graham et al. who show that MCL is not restricted to monocytes and macrophages, but it is also expressed on the surface of neutrophils. We also confirm its role in phagocytosis and function as an activating receptor through the association with the adaptor protein FcεRIγ.

## Materials and Methods

### Ethics Statement

Experimental animal protocols adhered to conventional ethical standards, followed the 3Rs principle and were approved by the Norwegian Research Animal Committee (protocol numbers 09.1170, 09.1555, and 11.3475).

### Animals

Animals were maintained under conventional rearing conditions in individually ventilated cages. BALB/c mice were bought from Harlan. DA.NKCB (NK complex from PVG) [13] and DA.APLEC-R1 (APLEC complex from PVG) congenic rats were generated and maintained in our animal facility. Animals were terminated by CO_2_ narcosis and asphyxiation. Lung and liver perfusions were conducted under anesthesia utilizing Hypnorm/Dormicum (both 5 mg/ml).

### Cell Lines and Primary Cells

BWZ.36 cells [14] were a kind gift from Dr N. Shastri (Department of Molecular and Cell Biology, University of California, Berkeley, USA). The BWN3G cell line was established in our lab by transfecting BW5147 cells with an NFAT-EGFP reporter construct [15]. Platinum-E (Plat-E) cells were purchased from Cell Biolabs [16]. The rat myeloid cell line RMW originated from PVG rat spleen as a side-product during the generation of an *in vitro*-passageable variant of the *in vivo*-restricted Roser leukemia (a rat T cell leukemic cell line) [17]. All other cell lines were purchased from ATCC. Plat-E cells were maintained in complete RPMI (RPMI 1640 supplemented with 10% FCS, 1% antibiotic-antimycotic, and 1 mM sodium pyruvate; Invitrogen) supplemented with 1 µg/ml puromycin and 10 µg/ml blasticidin (InvivoGen). 293T, BWZ.36 and BWN3G cell lines were grown in complete RPMI. Transduced cells were maintained in complete RPMI with 2 µg/ml puromycin. Stably transfected CHO cells were grown in complete RPMI supplemented with 1 mg/ml G-418 sulfate (PAA laboratories).

Blood was collected from the right atrium in heparinized syringes (heparin from LEO Pharma). Erythrocytes were lysed with RBC Lysis Buffer (BioLegend) according to the manufactureŕs instructions. Bone marrow tissue was carefully flushed out of femurs with ice-cold PBS, and red cells lysed as above. Sterile peritonitis was induced with intraperitoneal injections of zymosan (InvivoGen) (dose 4 mg/kg in PBS). PBS was employed as control. Injections were given under isoflurane anesthesia (Forene, Abbott). Peritoneal lavage was performed with cold PBS/5 mM EDTA 24 hours after injection. Cells were processed immediately after collection for flow cytometry staining and analysis. Organs were collected on ice-cold PBS and processed immediately. Spleen was digested with 2 µg/ml collagenase D (Roche) in RPMI 1640 with 1% FCS for 30 min at 37°C. Lungs were removed, washed thoroughly in PBS/5 mM EDTA, and digested with 2 µg/ml collagenase D in RPMI 1640 with 5% FCS for 30–90 min at 37°C until tissue disaggregation occurred. Resident peritoneal macrophages were isolated for *in vitro* culture using peritoneal lavage. Cells were centrifuged, resuspended in complete RPMI and allowed to adhere to plastic dishes for 2 h. Non-adherent cells were removed by washing, and remaining cells were cultured overnight in either M-CSF (20 ng/ml), IL-4 (20 ng/ml) or IFNγ (20 ng/ml) plus LPS (10 ng/ml) (cytokines were from Peprotech, LPS from InvivoGen). Cells were analyzed using flow cytometry 24 hours following the addition of cytokines.

### Generation of Constructs and Cell Lines

An expression construct consisting of the full-length rat MCL open reading frame followed by a C-terminal FLAG tag was generated in pEMCV-SRα and it was used for the transfection of CHO and 293T cells. Stable CHO transfectants were produced by electroporating cells, followed by subcloning and selection in complete RPMI supplemented with 1 mg/ml G-418 sulfate. 293T cells were transiently transfected using polyethyleneimine “Max” MW 25,000 (PEI) (Polysciences) [18].

A chimeric expression construct consisting of the cytoplasmic region of mouse CD3ζ followed by the transmembrane region of rat Killer cell lectin receptor H1 (KLRH1), the extracellular region of rat MCL and a C-terminal FLAG tag was generated in the retroviral vector pMXpuro. A similar chimeric construct was generated with rat Mincle. Plat-E cells were transfected with these vectors using PEI.

Cells were kept at 32°C and 5% CO_2_. PEI-containing medium was replaced on day 2 with fresh medium. On days 3, 4 and 5, virion-containing supernatants were harvested, centrifuged and filtered through a 0.45 µm syringe filter. Plat-E cells were analyzed by using flow cytometry to assess transfection efficiency. Viral particles were concentrated using polybrene and chondroitin sulfate complexation [19]. Before infection of BWZ.36 and BWN3G cells, polybrene was added to a final concentration of 4 µg/ml. Virions were added to the cells, and incubated at 32°C for 48 h. Transduced cells were cultured in puromycin selection medium (2 µg/ml) at 37°C. Cells were expanded and surface expression of receptors of interest analyzed by using flow cytometry.

### Generation of a Monoclonal Antibody against Rat MCL

Female BALB/c mice were immunized by injecting into the peritoneal cavity BWZ.36 cells expressing the chimeric MCL construct. Splenocytes were fused with NS-0 myeloma cells, and the hybridoma supernatants were screened for reactivity towards the extracellular region of rat MCL using the BWZ.MCL cells in a LacZ colorimetric assay as described previously [20]. After subcloning, one positive hybridoma clone was selected, isotyped (IgG_2a_ κ; kit from AbD Serotec) and named WEN42. WEN42 mAb was purified from hybridoma supernatant by protein G affinity chromatography (HiTrap Protein G HP column, GE Healthcare Life Sciences) and conjugated to NHS-Alexa Fluor 488 (A488) or -Alexa Fluor 647 (A647) (Invitrogen) according to the manufacturer’s instructions.

### Flow Cytometry

Cells (0.5–1×10^6^) were incubated for 15 min in 50 µl of PBS containing 10% rat serum and 10 mM NaN_3_ to block Fc receptors, before adding 50 µl of specific antibody (to a final concentration of 2 µg/ml) in PBS/1% FCS/10 mM NaN_3_ and incubating at 4°C for 20 min. Cells were then washed and analyzed by flow cytometry (FACSCanto II or FACSCalibur instruments, BD Biosciences). Fluorochrome conjugated mAbs to the following rat antigens were used: CD3 (clone G4.18-PE and -FITC), NKR-P1A (clone 10/78-PE and -FITC), CD8α (clone OX-8-PE), CD45RABC (clone OX-33-PE), granulocytes (clone HIS48-FITC) (BD Pharmingen); TCR γδ (clone V65-PE), CD80 (clone 3H5-PE), CD86 (clone 24F-PE), CD45RC (clone OX-22-A647), CD103 (clone OX-62-A647) (BioLegend); CD163 (clone ED2-FITC), CD169 (clone ED3-A488), MHC class II (clone OX6-FITC) (AbD Serotec). FcεRIα (clone 4H7.1) (Upstate Biotechnology). Monoclonal Abs towards rat CD172a (SIRPα, clone OX-41-biotin), CD11b/c (clone OX-42-A488 and -biotin), CD4 (clone W3/25-A488 and -biotin), MCL (clone WEN42-A647) were purified from hybridomas cultured in our laboratory, and conjugated to fluorochromes. Streptavidin-PE (Jackson ImmunoReseach Laboratories) was used for detection of biotinylated mAbs. Propidium iodide was used to identify dead cells. Appropriate isotype control conjugates were purchased from eBiosciences. Gating strategy is shown in supporting information Figure S2.

### Ligand Screening in Fungi

Transduced BWZ cells expressing the extracellular region of rat MCL were co-cultured with a panel of 20 fungal samples including yeasts and moulds (17 fungal species, Figure S3). Reporter cells (1×10^5^) were cultured for 18 h at 37°C in a volume of 200 µl of complete RPMI with heat-inactivated (30 min at 121°C) fungi at a ratio of 1∶10 (reporter:fungi). Cells were analyzed for β-galactosidase activity using a colorimetric assay. Fungi were grown using standard media and conditions at the Department of Microbiology, Oslo University Hospital.

### Immunohistochemistry

Frozen splenic sections (10 µm) were fixed in acetone, washed in PBS and incubated with primary antibodies towards rat CD169 (clone ED3), MHC class II (clone OX-6) or MCL (clone WEN42). mAb towards human MHC class I (clone W6/32, IgG_2a_) was used as an isotype control for WEN42. Peroxidase-conjugated rabbit anti-mouse IgG (Sigma-Aldrich) was pre-absorbed against tissue homogenates from rat lymph nodes and used as secondary antibody diluted 1∶1000 in PBS with 2% rat serum. Peroxidase activity was demonstrated with 3-3′-diaminobenzidine tetrahydrochloride (Sigma-Aldrich).

### Immunoprecipitation and Western Blot

CHO cells transfected with MCL were lysed in cold lysis buffer containing 25 mM Tris-HCl, pH 7.5, 150 mM NaCl, 1X Halt Protease and Phosphatase Inhibitor Cocktail EDTA-free (Thermo Fisher Scientific) in the presence of 0.1 mM CaCl_2_ and 1 mM MgCl_2_. CHO cells were lysed in lysis buffer containing n-octyl β-D-glucopyranoside (Sigma-Aldrich) and Triton X-100 (50 mM and 0.1% final concentrations respectively). RMW cells were lysed either in 50 mM n-octyl β-D-glucopyranoside and 0.1% Igepal CA-630 (Sigma-Aldrich) or 0.05% digitonin (Calbiochem). Criterion 12.5% and 15% Tris-HCl gels (Bio-Rad Laboratories) were used for SDS-PAGE protein separation under non-reducing and reducing (+5% 2-mercaptoethanol) conditions respectively. Samples were transferred onto Immobilon P polyvinylidene difluoride membranes (Millipore). Membranes were blocked in TBS/0.1% Tween 20/3% skimmed milk. Monoclonal Abs used for immunoprecipitation were: anti-MCL (WEN42), anti-human MHC class I (clone W6/32, isotype IgG_2a_), anti-FLAG (clone M2, Sigma-Aldrich). Antibodies used for immunoblotting were: polyclonal rabbit anti-FLAG (Sigma-Aldrich), anti-MCL-biotin (WEN42) and polyclonal rabbit anti-FcεRIγ (Upstate Biotechnology). Secondary antibodies used were goat anti-rabbit IgG-HRP and for biotinylated antibodies, streptavidin-HRP (Jackson ImmunoReseach Laboratories).

For pervanadate treatment, 1×10^7^ RMW cells were stimulated with 0.1% hydrogen peroxide and sodium orthovanadate (1 mM) in PBS at 37°C for 5 min prior to lysis. Membranes were blocked in TBS/0.1% Tween 20/3% BSA. For immunoblotting, anti-phosphotyrosine (clone 4G10, produced in-house) was used. Goat anti-mouse IgG-HRP (Jackson ImmunoReseach Laboratories) was used as secondary antibody. Detection by chemiluminescence was performed with Supersignal West Pico Chemiluminescent Substrate (Thermo Fisher Scientific).

### Electron Microscopy

RMW cells were detached from flasks with PBS/5 mM EDTA and fixed for 10 min with 2.5% glutaraldehyde followed by incubation in 1% OsO_4_ for 20 min. After washing thoroughly with PBS, 100 µl of a 5×10^6^ cells/ml cell suspension was applied into the top layer of an eppendorf tube containing 500 µl agar at 37°C. Cells were spun at 2000 rpm for 2 min at 37°C. Agar was allowed to solidify at 4°C before embedding in Durcupan (Fluka, Sigma-Aldrich). Ultrathin sections were prepared with a Reichert ultramicrotome and mounted on nickel grids for transmission electron microscopy (TEM) examination in a Fei Tecnai 12 microscope.

### Phagocytosis Assay

To label beads, 10 µl of NeutrAvidin-conjugated yellow-green fluorescent 1 µm microspheres (FluoSpheres, Invitrogen) were washed 3 times with PBS/0.5% BSA and then incubated with 5 µg of biotinylated antibody in 360 µl PBS/0.5% BSA for 30 minutes, rotating at 4°C. After washing three times beads were resuspended in 900 µl of PBS/0.5% BSA. RMW cells were resuspended at 1×10^7^ cells/ml in cRPMI supplemented with 10% rat serum and incubated at 4°C for 30 minutes to block Fc receptors. Cells were then plated at 1×10^6^ cells per well in 24-well plates. After addition of 100 µl of bead suspension to each well, plates were centrifuged at 500×g for 3 minutes, and then incubated at 37°C for one hour. Cells were washed twice in ice-cold PBS/1% FCS/0.1 mM NaN_3_, and then bead counterstaining performed by incubation with DyLight594-streptavidin (Jackson ImmunoReseach Laboratories) to identify non-internalized beads. Cells were then analyzed by imaging flow cytometry on an ImageStream X (Amnis). After gating on focused, single cells, cells with internalized beads could be identified by plotting bead-fluorescence against Dylight 594 fluorescence. Phagocytic ratio was defined as the ratio (bead-binding cells with internalized beads)/(bead-binding cells with no internalized beads). Analysis was performed using IDEAS software (Amnis).

## Results

### Generation of a Monoclonal Antibody towards Rat MCL

**Figure 1:**
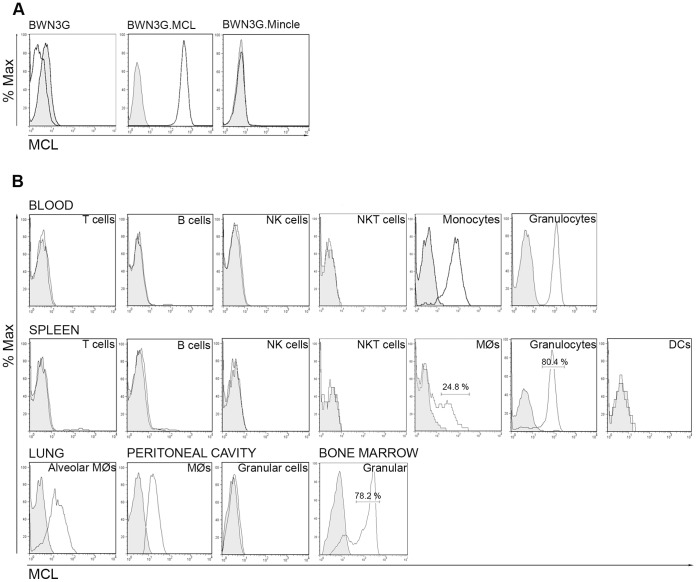
Rat MCL is expressed on monocytes, macrophages and granulocytes. A, mAb WEN42 is specific for MCL. The specificity of WEN42 was tested in flow cytometry analysis of BWN3G cells stably expressing rat MCL or Mincle. **B,** Flow cytometry analysis of rat MCL surface expression on different cell types isolated from different organs, as indicated. Histograms display cell subsets gated as described in the materials and methods section. Black line histograms: MCL. Filled grey histograms: isotype control.

As no antibody against rat MCL was commercially available, we produced our own in-house monoclonal anti-MCL antibody. BWZ cells virally-transduced with a chimeric protein consisting of the extracellular sequence of MCL, the transmembrane domain from killer cell lectin receptor H1 (KLRH1) and the cytoplasmic segment of the T-cell surface glycoprotein CD3 zeta chain were used for both immunization and screening as previously described [20]. Among the selected clones that showed high and consistent antibody production, we obtained three antibodies that bound specifically to rat MCL. One of these, WEN42, gave consistently better staining in flow cytometry, worked for immunoprecipitation and Western blot, and was used for all studies described herein. This antibody bound specifically to BWN3G cells expressing MCL (BWN3G.MCL), but not to the parent cells, or to cells expressing Mincle (BWN3G.Mincle) (Figure 1A). In addition, WEN42 did not bind to cells transfected with human MCL, or with other APLEC receptors (Figure S1).

### Cellular Expression of MCL Receptor in Rat Organs

Expression of MCL on subsets of immune cells isolated from different organs was analyzed using flow cytometry. We detected MCL expression on blood monocytes and neutrophils, but no expression was observed on T cells, B cells, NKT cells or NK cells. In the spleen, MCL expression was limited to macrophages and neutrophils. Spleen OX62**^+^** dendritic cells, T cells, gamma delta T cells and B cells did not express MCL. Resident peritoneal macrophages expressed MCL, but granular cells from the peritoneal cavity (mainly eosinophils and mast cells) were negative (Figure 1B). In the lung, alveolar and interstitial macrophages could be differentiated based on expression of SIRP-α (alveolar macrophages) or CD163 (interstitial macrophages) [21]. Alveolar macrophages were MCL^+^ (Figure 1B) whereas interstitial macrophages showed no expression (data not shown). In the bone marrow, a majority of the granular (SSC^high^) cell fraction expressed MCL. We were unable to detect expression of MCL on cells from the thymus, lymph nodes, small intestine, liver or Peyer’s patches (data not shown).

**Figure 2:**
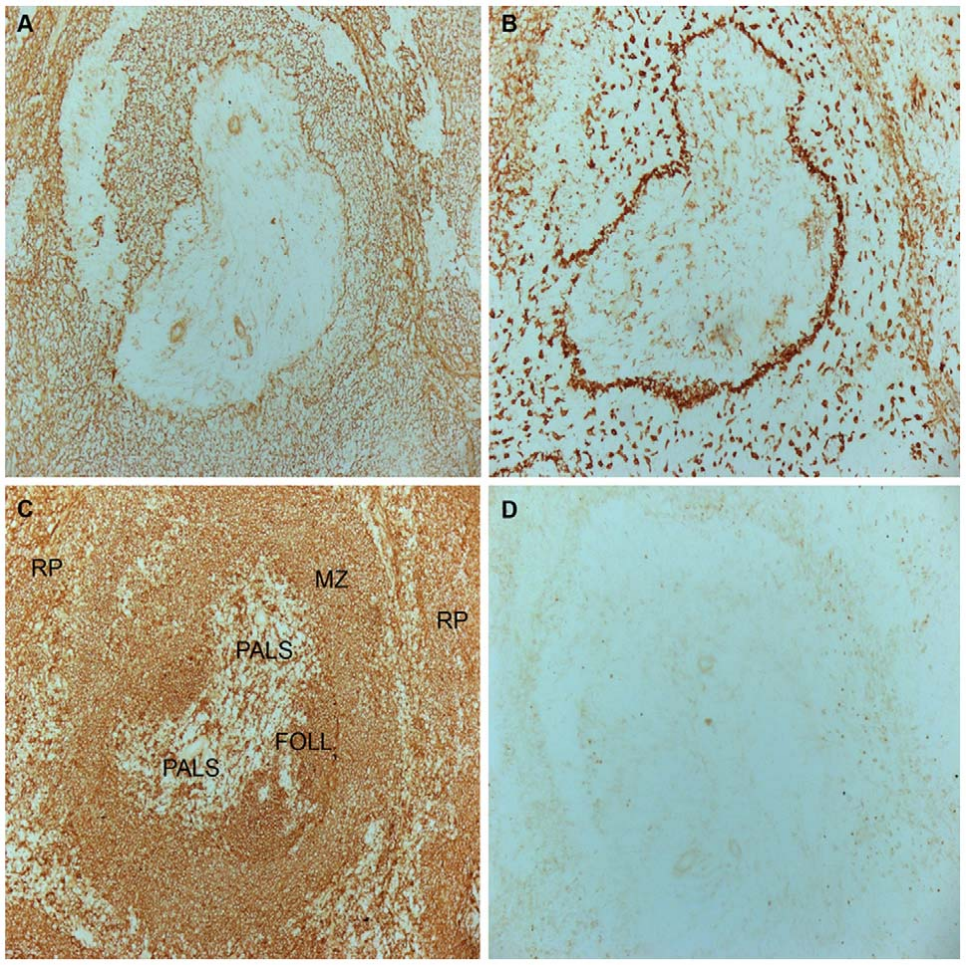
Immunostaining of rat spleen. Serial-cut frozen sections were stained with mAbs towards **A,** rat MCL **B,** rat CD169 **C,** rat MHC class II **D,** human MHC class I (negative control) and visualized with peroxidase-conjugated secondary antibody and DAB substrate. RP: red pulp. PALS: periarteriolar lymphoid sheath. FOLL: follicle. MZ: marginal zone.

Because the WEN42 mAb only stained a fraction of macrophages in splenic cell suspensions, we investigated the localization of MCL^+^ and MCL**^–^** cells in the spleen by immunohistochemistry. With WEN42, there was little or no staining of the white pulp (follicles and periarteriolar lymphoid sheaths, PALS), but we could observe distinct staining of cells in the marginal zone (MZ), probably MZ macrophages, including the thin marginal sinus layer of CD169^+^ MZ metallophilic macrophages at the border between the MZ and the white pulp. There was also abundant, although weaker, staining of the red pulp, probably red pulp macrophages (Figure 2).

### Zymosan Upregulates Cell Surface Expression of MCL on Activated Peritoneal Macrophages

**Figure 3:**
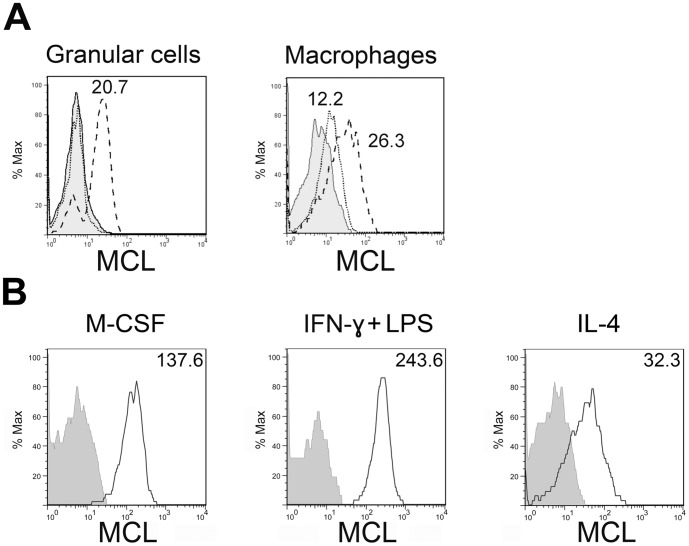
Regulation of MCL surface expression under the effect of different stimuli. **A,** Rats were injected intraperitoneally with zymosan, peritoneal cells were obtained after 24 h and stained with mAbs for flow cytometry analysis. Discontinuous line histogram shows staining in the zymosan treated group, dotted line histogram shows MCL staining in the PBS control group. Histograms display MCL expression on granular cells (including mast cells, eosinophils and neutrophils) and macrophages (MΦ) as indicated. The MCL^+^ fraction of granular cells likely reflects influx of neutrophils from the blood. MFI values are shown, calculated as the median of the fluorescence intensity. **B,** Resident peritoneal macrophages were cultured overnight with the indicated substances. Solid line histogram shows MCL staining. Grey filled histograms correspond to isotype controls.

Receptor expression under inflammatory conditions was examined by inducing sterile peritonitis with injections of zymosan in the peritoneal cavity of rats. 24 h after treatment, cells were collected by lavage of the peritoneal cavity and analyzed by flow cytometry. In the zymosan treated animals, MCL positive granulocytes appeared, corresponding to an expected influx of neutrophils. MCL expression was in addition increased on macrophages (Figure 3A). These were isolated from the peritoneum, maintained *in vitro* either in M-CSF, IFNγ plus LPS, or in IL-4. MCL was clearly increased when exposed to IFNγ and pro-inflammatory stimuli from G**^–^** bacteria, but it decreased following culture in the presence of anti-inflammatory IL-4 (Figure 3B). Taken together, these data suggest that MCL plays a role in inflammatory responses to microorganisms.

### MCL is Expressed by RMW, a Rat Myeloid Cell Line

**Figure 4:**
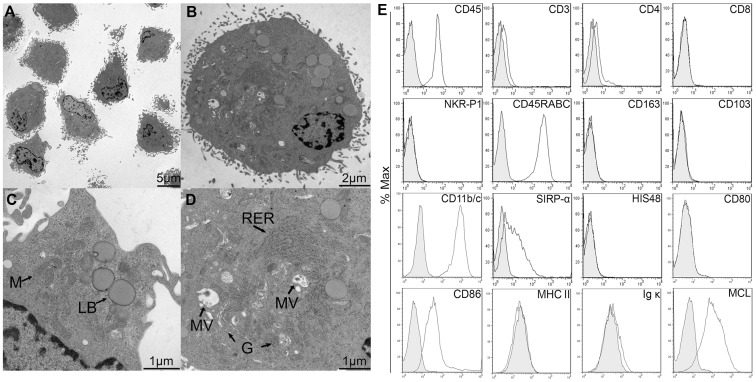
RMW is a rat myeloid cell line. A–D TEM ultrastructure of RMW cells. M: mitochondria. LB: lipid bodies. RER: rough endoplasmic reticulum. MV: multivesicular endosomes. G, Golgi. **E**, Flow cytometry analysis of RMW surface markers. Solid lines show the indicated surface markers; grey filled histograms correspond to isotype control.

The RMW cell line was derived by *in vitro* culture of PVG rat splenocytes as described [17]. Whereas the rat macrophage cell line R2 [22] did not express MCL (data not shown), RMW cells were MCL^+^ by flow cytometry. This cell line was considered a useful tool for further studies and was therefore characterized more thoroughly. Transmission electron microscopy analysis of the ultrastructure of RMW cells (Figure 4 A–D) showed a ruffled plasma membrane, suggesting a role for sensing and probing the extracellular environment. The nucleus was eccentrically located and indented, with a prominent nucleolus in many cells. The cytoplasm contained several mitochondria, and in most cells varying modest amounts of rough endoplasmic reticulum. Golgi complexes were distinct but not particularly prominent. Cytoplasmic vesicular structures were observed, including multivesicular bodies, and in some cells large structures with morphological characteristics of lipid bodies.

Analysis of flow cytometry data showed that RMW cells expressed typical myeloid markers (CD11b/c, SIRP-α, CD86), but lacked markers of T cells (CD3), NK cells (NKR-P1), dendritic cells (CD103) or granulocytes (HIS48) (Figure 4E).

### Biochemical Characterization of MCL

**Figure 5:**
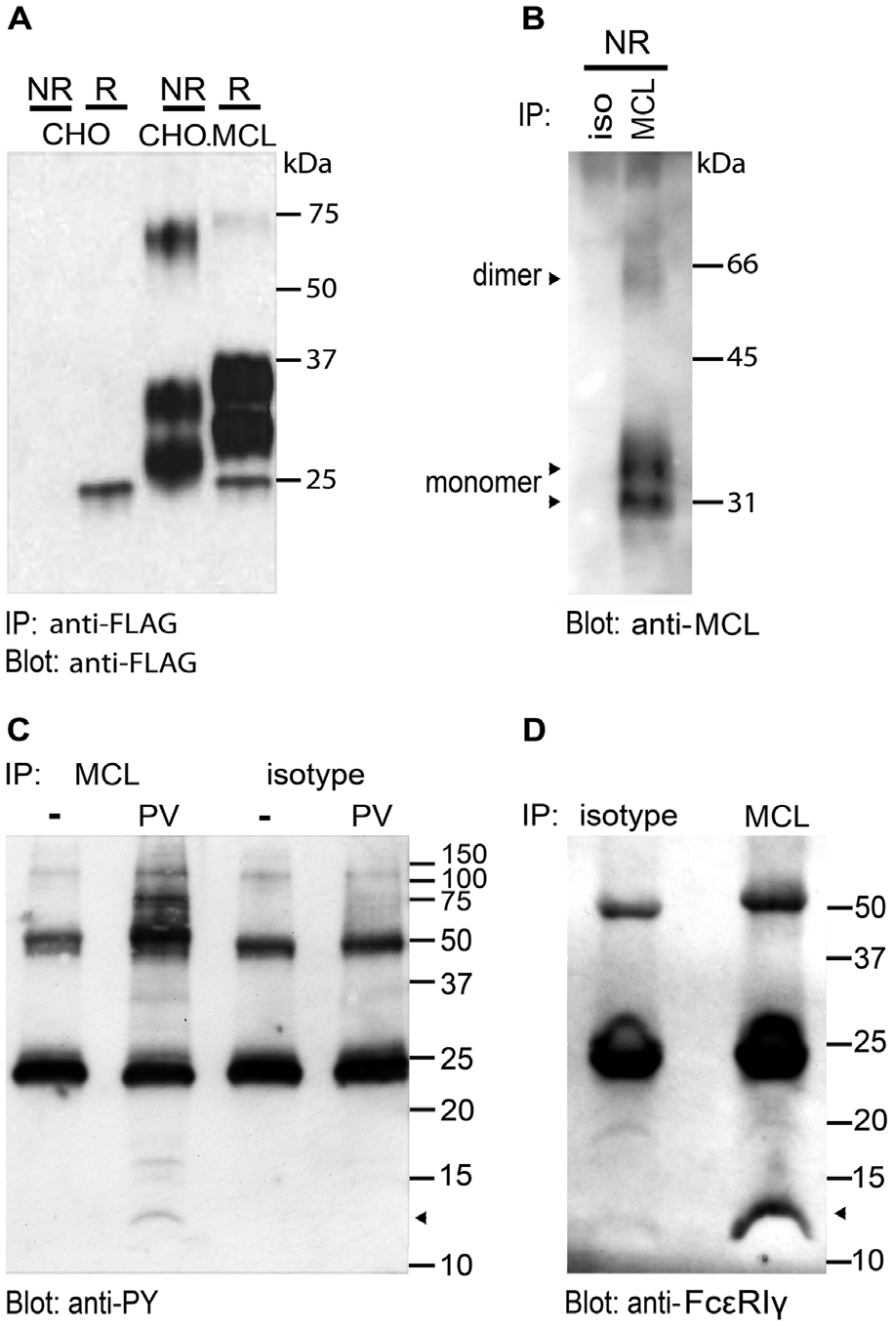
Biochemical analysis of rat MCL. A, Western blot analysis of anti-FLAG immunoprecipitate from CHO cells stably transfected with FLAG-tagged rat MCL. Lysate from parental CHO cells was used as control. Anti-FLAG mAb M2 was used for immunoprecipitation; blotting was performed with a rabbit polyclonal anti-FLAG antibody. **B,** Western blot analysis of anti-MCL immunoprecipitate from non-transfected RMW cells. Biotinylated anti-MCL mAb WEN42 was used for immunoblotting. **C,** Rat MCL co-precipitates with tyrosine phosphorylated molecules of different molecular weight. RMW cells were untreated (−) or treated with pervanadate (PV) before lysis, immunoprecipitated with anti-MCL mAb and subjected to Western blot analysis with an anti-phosphotyrosine mAb. **D,** Anti-FcεRIγ Western blot analysis of anti-MCL immunoprecipitate from unstimulated RMW cells. Iso: isotype control mAb. IP: immunoprecipitation. NR: non-reducing conditions. R: reducing conditions. Apparent molecular weight in kDa is indicated.

Rat MCL is a protein of 218 amino acid residues with predicted molecular weight of 25.3 kDa. It contains two putative N-linked glycosylation sites and two disulphide bonds involved in the stabilization of the C-type lectin-like domain. Stably transfected CHO cells were examined for possible formation of MCL multimers by Western blot analysis. In accordance with previously published results about human and mouse MCL [9], [11], the anti-rat MCL mAb reacted with bands of approximately 30 and 35 kDa in SDS-PAGE under reducing conditions. The ∼10 kDa increase over the predicted weight of the naïve MCL polypeptide most likely reflects glycosylation, and the occurrence of two bands is likely due to the presence of incompletely processed intracellular protein forms. Under non-reducing conditions, MCL migrated as a band of approximately 70 kDa, indicating that MCL exists as a disulfide-linked homodimer (Figure 5A). However, MCL was also found to migrate as a monomer under non-reducing conditions. Bands of similar size were also detected in RMW cells, suggesting that this receptor also exists as a single chain or forms non-covalently linked multimers. (Figure 5A, B). Following pervanadate treatment, a number of tyrosine phosphorylated proteins co-precipitated with MCL from RMW cells (Figure 5C), including a band of approximately 12 kDa, consistent with the expected size of FcεRIγ. A band of 12 kDa was also detected with specific antibody towards FcεRIγ in anti-MCL immunoprecipitation experiments, using non-stimulated RMW cells. Together, these data indicate that MCL associates with the activating transmembrane adaptor protein FcεRIγ, suggesting an activating function for MCL (Figure 5D).

### RMW Cells Phagocytose MCL-antibody-coated Beads

**Figure 6:**
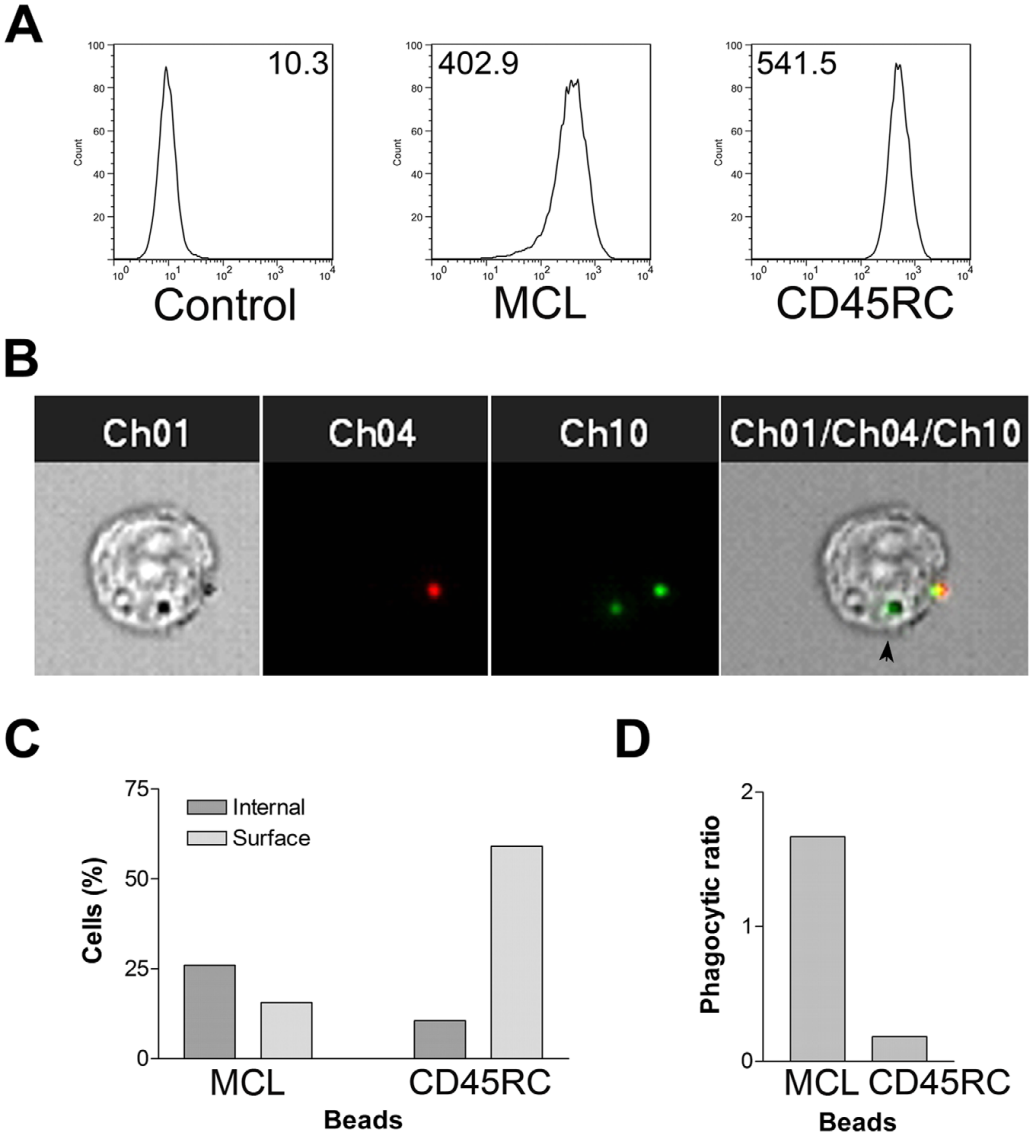
Phagocytosis of anti-MCL-coated beads. A, Comparative surface expression of the receptors, numbers represent median fluorescence intensity. **B.** Imaging flow cytometry analysis of a RMW cell showing a cell interacting with two beads. Channel 1 shows visible light image; bead-fluorescence is shown in channel 10, with DyLight-594 counterstaining in channel 4. Black arrowhead shows an internalized bead (green fluorescence) **C**. Percentage of cells with internalized beads, or with only surface-bound beads. **D.** Efficiency of phagocytosis expressed as a ratio of cells phagocytosing beads to cells with only surface-bound beads. 10,000 cells events were collected.

To determine whether MCL could play a role in phagocytosis, we coated fluorescent beads with antibodies to MCL or to CD45RC. Analysis of flow cytometry data showed that these antigens were expressed at similar levels on the surface of RMW cells (Figure 6A). After incubating beads with RMW cells for 1 h, we counterstained to differentiate internalized and phagocytosed beads, and analyzed using imaging flow cytometry. Figure 6B shows a sample image demonstrating an internalized bead and a surface bead on the same cell. We collected 10,000 cells and determined the percentage of cells that had internalized beads, and the percentage that only had cell surface beads. As shown in Figure 6C, the majority of cells that bound anti-MCL beads were able to phagocytose these beads, while the majority of cells that bound anti-CD45RC-beads did not. To correct for the fact that more cells bound to anti-CD45RC-beads than to anti-MCL-beads (Figure 6D), the data are expressed as a ratio of cells phagocytosing beads to cells with only surface-bound beads. These data indicate that MCL activates phagocytosis.

## Discussion

Our flow cytometry expression analysis confirms a restricted distribution of MCL on cells of the myeloid lineage, specifically, the monocytic and neutrophilic lineages. MCL is already present in some of the early stage precursors of these cells, as can be seen in the bone marrow analysis of granular cells. From our results we can conclude that neutrophil granulocytes and monocytes express MCL, whereas not all macrophage subpopulations display MCL on the surface. Interestingly, not all organ macrophages express MCL, as we could not find expression on cells from liver, lymph nodes, Peyer’s patches or interstitial lung macrophages.

It is well known that macrophages are a heterogeneous group of cells, with high plasticity that allows them to adapt to tissue environment [23], [24]. This is reflected in the diverse repertoire of surface receptors displayed by macrophages isolated from different tissues. For example, mouse macrophages of the peritoneal cavity express high levels of the phagocytic receptors CD11b and CD32, which indicate that the primary function of these macrophages is the detection of pathogens [25]. We have shown that rat resident peritoneal macrophages in a steady state express MCL, and that this expression is increased when sterile peritonitis is provoked by zymosan.

The lung contains two populations of macrophages, alveolar (AM) and interstitial macrophages (IM). Alveolar macrophages are well known for their phagocytic and microbicidal capacity, a characteristic indispensable in this organ where the cells encounter a vast amount of airborne microorganisms such as fungi, bacteria and viruses. At the same time, these cells may fulfill both pro- and anti-inflammatory roles, necessary for the homeostasis of this organ [26], [27]. Although IM have also been shown to mediate phagocytosis, they are not as effective as AM, but they are more efficient at processing antigens, expressing higher levels of MHC class II and C3 receptor [28]–[30]. The expression of MCL solely on alveolar macrophages could suggest a function for this protein in the recognition of microorganisms that enter the body via this route. In the spleen we also found differential MCL expression. Different subpopulations of tissue macrophages are located in the red pulp, white pulp, and the marginal zone, each with their own role [31]–[33]. Immunohistochemistry analysis showed that MCL expression is primarily on the marginal sinus marginal metallophilic macrophages between the white pulp and MZ, with expression also on MZ macrophages through the MZ and red pulp. Marginal zone macrophages are involved in the initiation of the innate immune responses through recognition of blood-borne microorganisms, while red pulp macrophages are primarily involved in the recycling of iron from aged and damaged erythrocytes, but can also recognize and eliminate microorganisms; again this is consistent with a role for MCL in pathogen recognition.

mRNA expression data from different studies show that MCL is upregulated under inflammatory conditions. Macrophages from C57BL/6 mice upregulate MCL following infection with *Klebsiella pneumoniae* [34]. Noriyuki Fujikado and colleagues generated two arthritis mouse models that develop rheumatoid arthritis spontaneously within 4 and 5 weeks of age. Gene expression profiling of whole joint homogenate from these two models reveals an upregulation of MCL [35]. In our study, we show that MCL is upregulated on peritoneal macrophages *in vivo*, following challenge with zymosan. Further, we demonstrate that exposure of these macrophages to IFNγ and the TLR-4 ligand LPS leads to up-regulation of MCL expression, while, conversely, culture with the anti-inflammatory cytokine IL-4 leads to down-regulation of MCL expression. This strongly suggests that MCL has its primary role in inflammatory situations.

Biochemical analysis of rat MCL expressed on stably transfected CHO cells suggests that the receptor may exist mainly as a monomer, with a smaller fraction present as homodimers. Western blot assays from RMW cells also demonstrated that rat MCL predominantly migrated as a single chain, with a smaller fraction present as a homodimer. This differs from other C-type lectin type II membrane receptors that are generally expressed as covalently linked multimers. To rule out that the presence of MCL in monomeric form was an artifact of our transfection systems, we performed immunoprecipitations with the mAb WEN42 and Western blot analysis of endogenously expressed MCL in the RMW cell line. These experiments also demonstrated the presence of MCL monomers under non-reducing conditions. The single-chain MCL observed in our experiments may represent monomeric MCL expressed on the cell surface. In support of this, human myeloid C-type lectin-like receptor (MICL) has been reported to be expressed as a heavily glycosylated monomer [36]. The observed single-chain MCL may also represent ER resident MCL that has not yet been folded properly, and therefore, is not expressed on the cell surface. Alternatively, rat MCL may form multimeric receptors that are not covalently linked. This would be similar to the C-type lectin-like receptor chains KLRE and KLRI that are expressed on the cell surface as non-covalently linked heterodimers [37]. Phosphotyrosine analysis of proteins co-precipitating with MCL from RMW following pervanadate treatment reveals a number of tyrosine-phosphorylated associated proteins, including a protein of approximately 12 kDa, consistent with the adaptor protein FcεRIγ. However, we have not been able to detect a direct association of MCL and FcεRIγ in transfected cells (data not shown), which suggests that such an association may be indirect. Nevertheless, this suggests that MCL is likely an activating receptor, consistent with the fact that it mediates phagocytosis of antibody-coated beads.

Whether or not the MCL carbohydrate recognition domain (CRD) plays a role in identifying pathogen-associated molecular patterns (PAMPs) is not known. We have screened a panel of 15 fungal species, including in total 17 yeast and 3 mould strains, for ligands for MCL, but have not seen any evidence of ligands on the strains tested (Figure S3). The closely related APLEC receptor, Mincle, has been shown to preferentially recognize a limited subset of fungi, in particular Malassezia sp. [4], in a CRD-dependent manner. It is possible that we have simply not screened the fungi that are recognized by MCL. Alternatively, MCL may recognize other pathogens that we have not screened. Tsui-Ling Hsu et al. submitted a panel of human receptor-Fc fusion proteins for glycan array analysis to the Consortium for the Functional Glycomics (CFG). Human MCL, among other APLEC receptors, was included. However, no conclusive data could be obtained for this chimera [38].

### Conclusions

In summary, we show that rat MCL is an activating receptor expressed on neutrophils and subsets of macrophages. MCL expression is increased *in vivo* after exposure to zymosan, while *in vitro* it is expressed preferentially on macrophages exposed to pro-inflammatory stimuli. This, together with its distribution on macrophage subsets, suggests that rat MCL is an inflammatory receptor that most likely plays a role in pathogen recognition and phagocytosis.

## Supporting Information

Figure S1 **Validity of staining of mAb WEN42 in transfected CHO cells and transduced BWZ cells.**
(TIF)

Figure S2 **Gating strategy for the analysis of flow cytometry data.** **A**, Blood. **B**, Spleen. **C**, Lung. **D**, Peritoneal cavity. **E**, Bone marrow. T cells were identified as CD3**^+^**; B cells as CD45RABC**^+^** cells. NK cells were identified as CD3**^–^** NKR-P1^bright^ and NKT cells as CD3**^+^** NKR-P1^dim^. Blood monocytes were gated as SSC^low^ CD4^dim^ cells. Granulocytes were identified as SSC^high^ His48**^+^** CD4**^–^**. Spleen macrophages were gated as SSC^low^ CD4^dim^. Spleen dendritic cells were gated as MHC class II**^+^** CD103**^+^**. Spleen γδ T cells were identified as CD3**^+^** TCR γδ**^+^**cells. Lung alveolar macrophages were gated as SSC^low^ OX41^+^ CD4^dim^ CD163**^–^** cells, interstitial macrophages as SSC^low^ CD4**^+^** CD163**^+^** cells. Peritoneal cavity granular cells were gated as SSC^high^ OX42^dim^ OX41^dim^ or alternatively as SSC^high^ OX42^dim^ CD4**^–^** cells. Peritoneal macrophages were gated as SSC^low^ OX42^bright^ OX41^bright^ or alternatively SSC^low^ OX42^bright^ CD4^dim^ cells. Peritoneal mast cells and basophils were identified as SSC^high^ FcεRIα**^+^**. Bone marrow cells were also separated on the basis of cytoplasmic granularity as SSC^high^ and SSC^low^.
(TIF)

Figure S3 **MCL receptor ligand screening in a panel of fungi.** Transduced BWZ.rMCL reporter cells (1×10^5^) were cultured for 18 h with heat-inactivated fungi at a ratio 1∶10 (reporter:fungi). A total of 17 fungal species were tested. Ligand recognition was analyzed using the colorimetric LacZ assay. Numbers in brackets refer to different laboratory samples.
(TIF)
