# Supplementary figures and images for: Rat Macrophage C-Type Lectin Is an Activating Receptor Expressed by Phagocytic Cells
Source: PLoS One. 2013 Feb 28;8(2):e57406. doi: 10.1371/journal.pone.0057406 (PMC3585393; doi:10.1371/journal.pone.0057406)

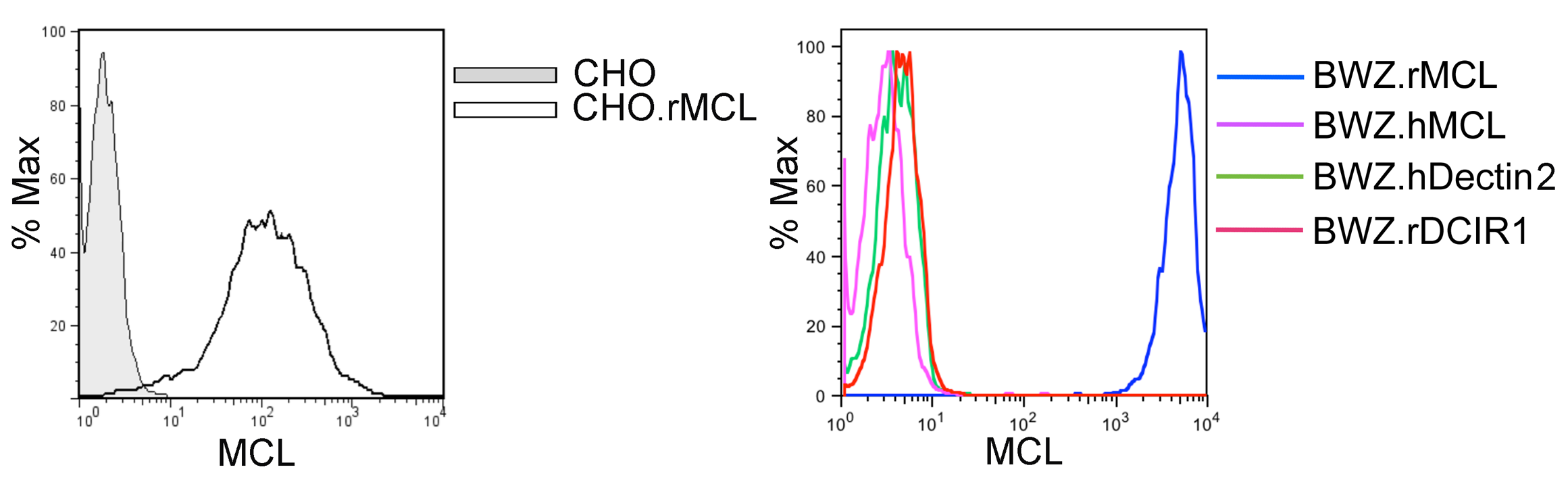

Supplement: Figure S1 — Validity of staining of mAb WEN42 in transfected CHO cells and transduced BWZ cells. (TIF) [file pone.0057406.s001.tif]

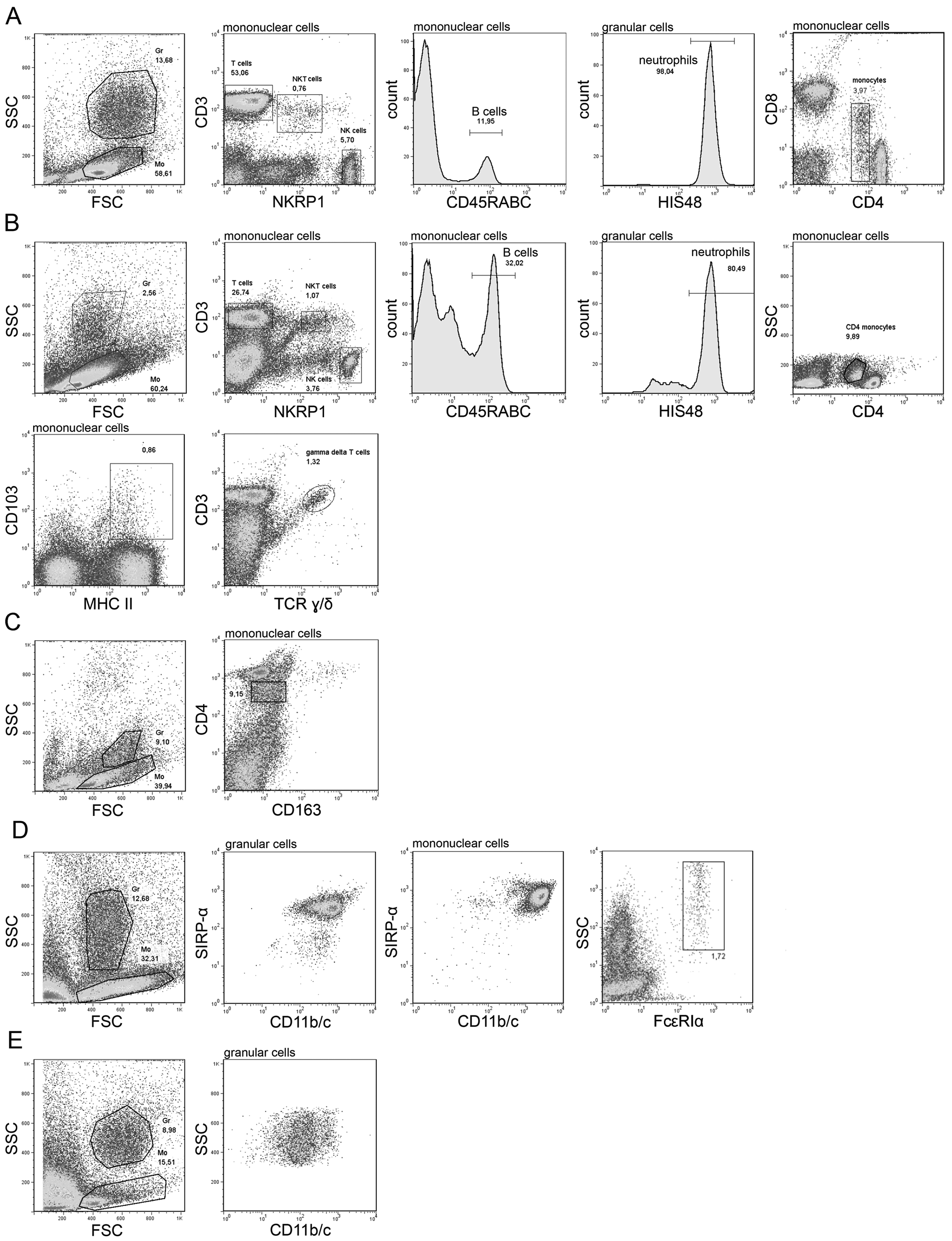

Supplement: Figure S2 — Gating strategy for the analysis of flow cytometry data. A, Blood. B, Spleen. C, Lung. D, Peritoneal cavity. E, Bone marrow. T cells were identified as CD3+; B cells as CD45RABC+ cells. NK cells were identified as CD3– NKR-P1bright and NKT cells as CD3+ NKR-P1dim. Blood monocytes were gated as SSClow CD4dim cells. Granulocytes were identified as SSChigh His48+ CD4–. Spleen macrophages were gated as SSClow CD4dim. Spleen dendritic cells were gated as MHC class II+ CD103+. Spleen γδ T cells were identified as CD3+ TCR γδ+cells. Lung alveolar macrophages were gated as SSClow OX41+ CD4dim CD163– cells, interstitial macrophages as SSClow CD4+ CD163+ cells. Peritoneal cavity granular cells were gated as SSChigh OX42dim OX41dim or alternatively as SSChigh OX42dim CD4– cells. Peritoneal macrophages were gated as SSClow OX42bright OX41bright or alternatively SSClow OX42bright CD4dim cells. Peritoneal mast cells and basophils were identified as SSChigh FcεRIα+. Bone marrow cells were also separated on the basis of cytoplasmic granularity as SSChigh and SSClow. (TIF) [file pone.0057406.s002.tif]

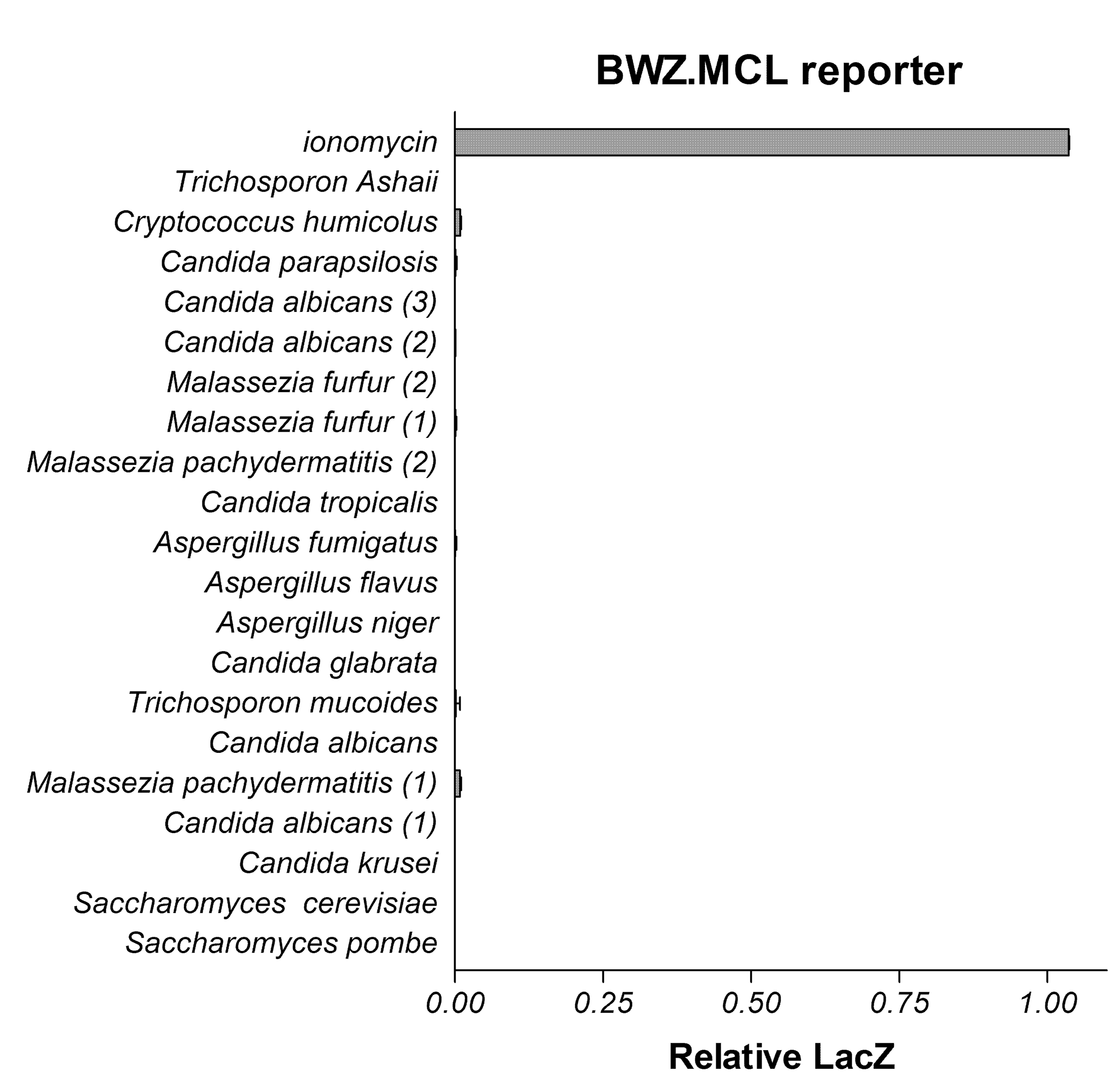

Supplement: Figure S3 — MCL receptor ligand screening in a panel of fungi. Transduced BWZ.rMCL reporter cells (1×10^5^) were cultured for 18 h with heat-inactivated fungi at a ratio 1∶10 (reporter:fungi). A total of 17 fungal species were tested. Ligand recognition was analyzed using the colorimetric LacZ assay. Numbers in brackets refer to different laboratory samples. (TIF) [file pone.0057406.s003.tif]
